# Supplementary material for: Distinct clonal evolution of B-cells in HIV controllers with neutralizing antibody breadth
Source: eLife. 2021 Apr 12;10:e62648. doi: 10.7554/eLife.62648 (PMC8041465; doi:10.7554/eLife.62648)
Supplement: Supplementary file 1. — CD4+ T-cell counts (cells/μl), viral loads (HIV RNA copies/ml) and neutralization titers (50% inhibitory dose [ID50], 1/x] from each individual against a panel of 11 tier 2 and 3 envelope (Env)-pseudoviruses and murine leukemia virus (MuLV)-pseudotyped virion control. The right column (breadth %) shows the % of viruses that are neutralized above background (3× MuLV control). Six individuals with neutralization of more than 90% of the panel classified as top-neutralizer (TN) while neutralization of less than 10% of the panel viruses classified as non-neutralizer (NN). [file elife-62648-supp1.docx]

| **Subject ID** | **CD4 cells** | **Viral Load** | **Tier 2 - QH0692.42** | **Tier 2 -SC422661.8** | **Tier 2/3 - PVO.4** | **Tier 2 -TRO.11** | **Tier 2 -AC10.0.29** | **Tier 2 -RHPA4259.7** | **Tier 2 -THRO4156.18** | **Tier 2 -REJO4541.67** | **Tier 2/3 -TRJO4551.58** | **Tier 2 -WITO4160.33** | **Tier 2 -CAAN5342.A2** | **3x MuLV** | **Breadth (%)** | **Neutralizer?** |
| --- | --- | --- | --- | --- | --- | --- | --- | --- | --- | --- | --- | --- | --- | --- | --- | --- |
| **330183** | 1038 | 3180 | 188 | 425 | 547 | 1175 | 1286 | 357 | 192 | 1883 | 598 | 3410 | 313 | 60 | 100 | TN |
| **280008** | 817 | 20 | 130 | 639 | 309 | 498 | 1462 | 287 | 346 | 851 | 279 | 134 | 207 | 84 | 100 | TN |
| **315504** | 436 | 1950 | 83 | 179 | 154 | 278 | 98 | 131 | 120 | 339 | 177 | 94 | 203 | 60 | 100 | TN |
| **622800** | 697 | 454 | 187 | 165 | 427 | 606 | 626 | 404 | 22 | 429 | 143 | 1348 | 217 | 111 | 91 | TN |
| **628655** | 847 | 273 | 56 | 158 | 356 | 498 | 143 | 198 | 511 | 379 | 224 | 92 | 170 | 90 | 91 | TN |
| **444154** | 778 | 20 | 71 | 202 | 48 | 170 | 160 | 94 | 79 | 275 | 114 | 156 | 71 | 63 | 91 | TN |
| **847041** | 1769 | 20 | 211 | 1150 | 423 | 655 | 64 | 212 | 115 | 1488 | 632 | 1115 | 176 | 147 | 82 | MN |
| **745577** | 796 | 15800 | 84 | 222 | 35 | 197 | 183 | 113 | 205 | 428 | 114 | 280 | 27 | 69 | 82 | MN |
| **939369** | 632 | 546 | 49 | 878 | 915 | 359 | 2533 | 600 | 402 | 37 | 45 | 286 | 156 | 117 | 73 | MN |
| **437105** | 749 | 1190 | 61 | 293 | 99 | 114 | 519 | 1045 | 71 | 493 | 277 | 65 | 161 | 93 | 73 | MN |
| **196203** | 828 | 24800 | 112 | 158 | 43 | 222 | 149 | 47 | 77 | 857 | 1351 | 718 | 70 | 93 | 64 | MN |
| **187940** | 638 | 219 | 292 | 384 | 728 | 335 | 509 | 330 | 108 | 78 | 86 | 139 | 207 | 240 | 55 | MN |
| **504350** | 925 | 466 | 64 | 115 | 311 | 247 | 52 | 1525 | 42 | 103 | 32 | 810 | 32 | 108 | 45 | MN |
| **447160** | 690 | 20 | 67 | 76 | 20 | 160 | 186 | 170 | 20 | 20 | 28 | 46 | 20 | 60 | 45 | MN |
| **701998** | 729 | 20 | 35 | 189 | 28 | 137 | 20 | 200 | 20 | 20 | 25 | 72 | 20 | 60 | 36 | MN |
| **785360** |  | 20 | 253 | 340 | 169 | 285 | 226 | 454 | 97 | 599 | 281 | 254 | 160 | 498 | 9 | NN |
| **534694** | 663 | 5450 | 60 | 45 | 27 | 21 | 81 | 55 | 20 | 32 | 32 | 20 | 189 | 108 | 9 | NN |
| **756587** | 661 | 20 | 35 | 70 | 20 | 33 | 20 | 20 | 20 | 26 | 49 | 20 | 20 | 60 | 9 | NN |
| **149812** | 938 | 20 | 20 | 20 | 20 | 20 | 20 | 20 | 20 | 20 | 20 | 20 | 20 | 60 | 0 | NN |
| **201441** | 700 | 267 | 57 | 20 | 32 | 37 | 46 | 20 | 56 | 20 | 20 | 90 | 20 | 186 | 0 | NN |
| **211774** | 537 | 687 | 20 | 20 | 20 | 20 | 20 | 20 | 20 | 20 | 20 | 20 | 20 | 60 | 0 | NN |
| **386576** | 602 | 132 | 52 | 69 | 90 | 80 | 48 | 113 | 118 | 42 | 65 | 40 | 46 | 129 | 0 | NN |
